# Locally-Actuated Graphene-Based Nano-Electro-Mechanical Switch

**DOI:** 10.3390/mi7070124

**Published:** 2016-07-19

**Authors:** Jian Sun, Manoharan Muruganathan, Nozomu Kanetake, Hiroshi Mizuta

**Affiliations:** 1 School of Material Science, Japan Advanced Institute of Science and Technology, Nomi 923-1211, Japan; mano@jaist.ac.jp (M.M.); s1330021@jaist.ac.jp (N.K.); 2 Advanced Device Laboratory, RIKEN, Wako 351-0198, Japan; 3 Nanoelectronics and Nanotechnologies Research Group, Faculty of Physical Sciences and Engineering, University of Southampton, High Field, Southampton SO17 1BJ, UK

**Keywords:** graphene, nano-electro-mechanical system, nano-electro-mechanical switch, nanofabrication

## Abstract

The graphene nano-electro-mechanical switches are promising components due to their outstanding switching performance. However, most of the reported devices suffered from a large actuation voltages, hindering them from the integration in the conventional complementary metal-oxide-semiconductor (CMOS) circuit. In this work, we demonstrated the graphene nano-electro-mechanical switches with the local actuation electrode via conventional nanofabrication techniques. Both cantilever-type and double-clamped beam switches were fabricated. These devices exhibited the sharp switching, reversible operation cycles, high on/off ratio, and a low actuation voltage of below 5 V, which were compatible with the CMOS circuit requirements.

## 1. Introduction

Nano-electro-mechanical (NEM) switches, utilizing electrostatic forces to mechanically deflect the active element into physical contact with an electrode, are of great interest for future logic devices, relays and sensors [1]. Graphene has an ultra-high Young’s modulus of 1 TPa, making it a promising candidate for future NEM applications. The graphene NEM contact switches showed minimized electrical leakage, sharp switching response, low actuation voltage, and a high on/off ratio [2,3,4,5,6,7]. In most of the reported works, the heavily-doped silicon substrate has been utilized as the actuation electrode. However, as a consequence, the graphene NEM switch suffers from the relatively large pull-in voltage, normally greater than 10 V [8]. It is mainly ascribed to the thick, inevitable dielectric gap in additional to a pure air gap in the practical globally-actuated graphene switch. Hence, there is a demand to include the local actuation electrode in order to reduce the pull-in voltage. However, the reported fabrication procedure is complicated, i.e., burying a local bottom gate under the graphene, and not compatible with the standard thin-film bottom-up fabrication process techniques [9]. Moreover, it is known that the cantilever-type suspended structure has a smaller spring constant, i.e., mechanical strength, than the double-clamped beam. A graphene cantilever, in principle, could much reduce the pull-in voltage based on the proper geometric design. However, we noticed just a few experimental studies on graphene cantilevers [10], and only one of them reported the cantilever-type graphene switch device [9], as its fabrication is much more challenging compared with the double-clamped beam.

In this work, we demonstrated the simple procedure to fabricate graphene NEM switches with both double-clamped beam and cantilever-type moving elements. By this method, the local top electrodes can be introduced for actuation. The fabricated devices were characterized with a two-terminal configuration, showing low pull-in voltages of less than 5 V. The results demonstrated the possibility of integrating such NEM switches with a conventional complementary metal-oxide-semiconductor (CMOS) circuit for future applications.

## 2. Experimental Details

### 2.1. Switch Fabrication

**Figure 1:**
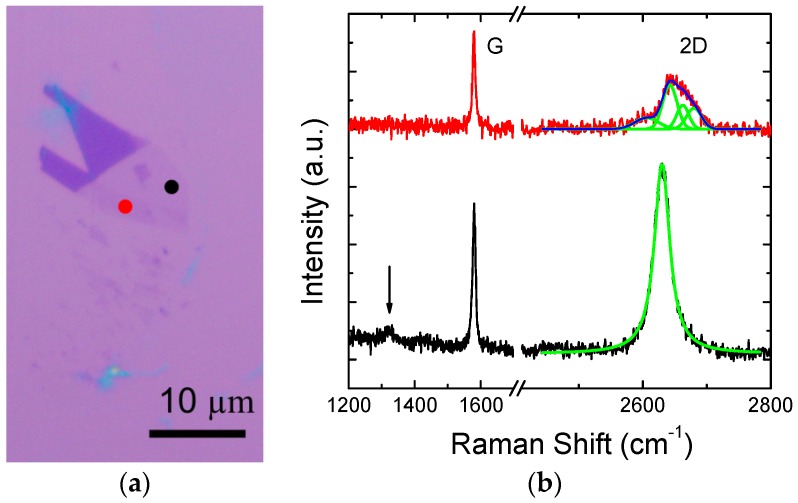
(**a**) Exfoliated graphene flake, black and red dots indicate the monolayer and bilayer regions, respectively; and (**b**) Raman spectra probed at locations marked as two color dots in (**a**). Green lines are the sub-peaks obtained from Lorentzian peak fitting. The arrow indicates the location of D band.

The fabrication process started with the mechanically-exfoliated monolayer graphene flakes on the p-doped silicon substrate covered with 300 nm thermal SiO_2_. The possible graphene flakes were firstly identified from their contrast to the substrate under an optical microscope. Figure 1a shows an exfoliated graphene flake with the various numbers of layers. Then Raman spectroscopy (HeNe laser: 633 nm excitation) was utilized to verify the actual number of layers of the exfoliated graphene [11]. Monolayer graphene was firstly identified by a larger intensity of the 2D band over the G band (Figure 1b). Moreover, its 2D band can be fitted with a single Lorentzian peak with a full width at a half-maximum of <30 cm^−1^. For the bilayer graphene, four Lorentzian sub-peaks are fitted to the 2D band. In principle, the exfoliated graphene should be intact from significant lattice defects. The mild D peak observed in the measured spectra are ascribed to the defective edges of the flake near the probed areas. 

**Figure 2:**
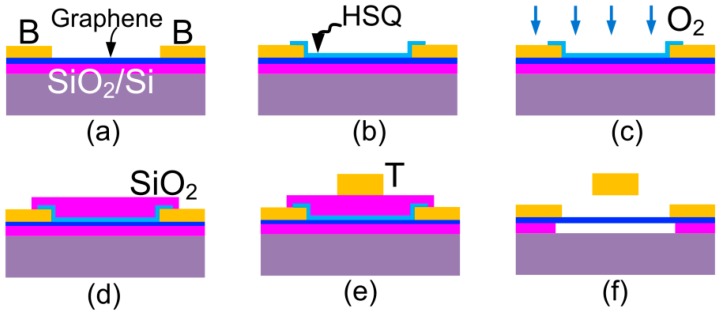
Schematics of fabrication procedure of the graphene NEM switch with local top actuation electrode. T and B denote top and bottom electrodes, respectively. (**a**) Graphene exfoliation and bottom electrodes definition; (**b**) HSQ spin coating and pattering; (**c**) graphene etching in oxygen plasma; (**d**) definition of SiO_2_ sacrificial layer; (**e**) definition of top electrode; (**f**) releasing graphene in HF.

**Figure 3:**
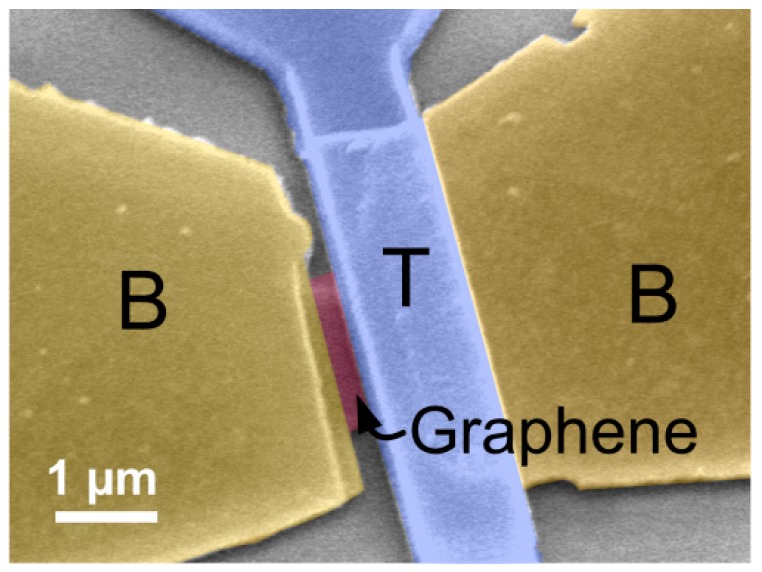
False color SEM image of a double-clamped beam graphene switch as-fabricated. T and B denote top and bottom electrodes, respectively.

Figure 2 sketches the fabrication process after exfoliation. (a) Firstly, the bottom contacts to graphene flakes were defined with a Cr/Au (5 nm/60 nm) stack following the electron-beam lithography (EBL), evaporation, and lift-off processes; (b) then, hydrogen-silsesquioxane (HSQ) resist was spun and patterned by electron beam lithography (EBL) into the desired shapes acting as the etching masks for the next step; (c) graphene was patterned by transferring the shapes from capping HSQ masks in the oxygen plasma environment in a reactive ion ether; (d) the SiO_2_ layer was evaporated and capped onto graphene/HSQ as a sacrificial layer; (e) the top actuation electrode was defined with Cr/Au (5 nm/200 nm); and (f) etch all of the oxide layers (HSQ, sacrificial SiO_2_, and SiO_2_ substrate) in the buffered hydrofluoric acid (1:5) to release graphene from the supporting substrate. Finally, the device was dried in a critical point drier to prevent surface tension-induced collapse of the suspended graphene. Figure 3 shows a typical fabricated switch.

### 2.2. Device Characterization

**Figure 4:**
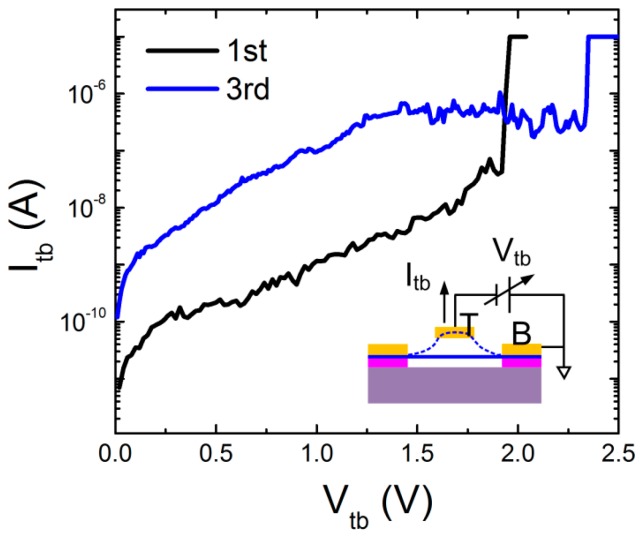
Electrical characterization of the switching performance of a double-clamped beam switch. The inset shows the two-terminal measurement configuration.

All of the fabricated graphene switches were characterized in the vacuum condition (~0.1 Pa) to reduce the impacts from ambient, such as the molecular absorption and capillary force due to moisture. The two-terminal configuration is used to investigate switch performance (lower inset of Figure 4), where the top electrode works as both the current drain and actuation electrode. A variable voltage *V*_tb_ was applied between top and bottom electrodes and swept from 0 V to higher voltages, which could electrostatically deflect the graphene upwards to the top electrode, while the current between two electrodes *I*_tb_ was monitored.

## 3. Results and Discussions

### 3.1. Double-Clamped Beam Switch

Figure 3 shows the scanning electron microscope (SEM) image of a fabricated switch with a double-clamped monolayer graphene beam and top actuation electrode. The graphene beam has a length *l* of 1 µm and width *w* of 2 µm. An air gap between the graphene and top electrode is clearly noticed. The gap thickness *g*_0_ is almost defined by the total thickness of HSQ and SiO_2_ sacrificial layer, which is ~180 nm in this device.

At the initial stage, an open circuit was expected between the top and bottom electrode, namely, the switch-off status. Hence, at the beginning of the *V*_tb_ sweeping, only leakage current of a pico-ampere level was measured. At ~1.92 V, we noticed an abrupt increase of current to the compliance value. This strongly indicates that graphene was physically pulled into the top electrode, as the electrostatic force applied by the top electrode balanced the mechanical restoring force in the graphene beam. In other words, the device was switched on. This actuation voltage is often termed as the pull-in voltage *V*_pi_. Comparing to those switches using doped Si substrates as global actuation electrodes [1,8], the much stronger electrostatic force generated by the local top electrode brings about the low *V*_pi_, benefiting from the well-controlled pure air gap. Moreover, the steep switching slope of ~15 mV/dec and on/off ratio of ~10^3^ was read from the measurement, highlighting the rapid switching response. Note, a low compliance current of 10 μA was set for this measurement, leading to a much underestimated on current. By assuming the on current in the microampere range, the accrual on/off ratio should be in the order of 10^5^.

The pull-in voltage of a double-clamped beam can be theoretically calculated as [8]:

(1) $$V_{\text{pi}}=\sqrt{\frac{8kg_{0}^{3}}{27\varepsilon Wl}},\;k=32Ew{\left(\frac{t}{l}\right)}^{3}$$

where *k* is the spring constant of the graphene beam, *E* is the Young’s modulus of graphene, *t*, *l*, *w* are the thickness, length, and width of the graphene beam, respectively, *ε* is permittivity, and *W* is the overlapping width of top electrode on graphene. With Equation (1) the pull-in voltage of the switch with a clean graphene, which has the thickness *t* of 0.34 nm, is estimated as 0.70 V. However, the contaminations and adsorbates on graphene are difficult to avoid after fabrication. By assuming a monolayer of water molecularly adsorbed on the graphene surface, it increases the beam thickness to ~0.7 nm; *V*_tb_ of 2.07 V is then calculated with Equation (1), which is close to the measurement. The current annealing process could be employed to remove the adsorbates [12].

We retracted the actuation voltage application immediately after the observation of pull-in to avoid the joule heating accumulation at the contact interface. This is known as the main reason of device failure, since it triggers the formation of chemical bonds between graphene and the contact metal and causes graphene sticking on contact [6]. Reversible switching operation was realized in this device. However, the slight fluctuation in pull-in voltage was observed; a *V*_tb_ of 2.3 V was measured in the third operation cycle. It is ascribed to the enhanced leakage current through the degraded substrate (read as the enhanced leakage current background), which effectively reduces the strength of the electric field flux at the graphene surface and, therefore, weakening the electrostatic force.

### 3.2. Cantilever-Type Switch

**Figure 5:**
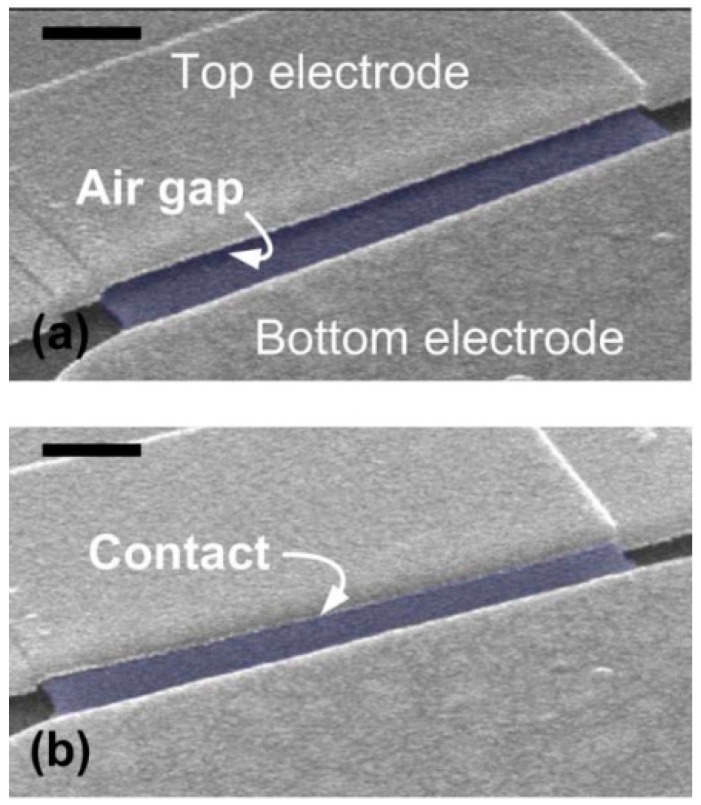
SEM images of a graphene cantilever-type switch with a local top actuation electrode (**a**) before and (**b**) after the switching operation. The initial air gap *g*_0_ between the graphene and the top electrode is about 140 nm. The graphene cantilever is artificially colored in light blue. The scale bars are 500 nm.

Figure 5a shows the SEM image of a few-layer (~10 layers) graphene cantilever switch. An air gap between the graphene and top electrode defined by the sacrificial layer is clearly noticed.

**Figure 6:**
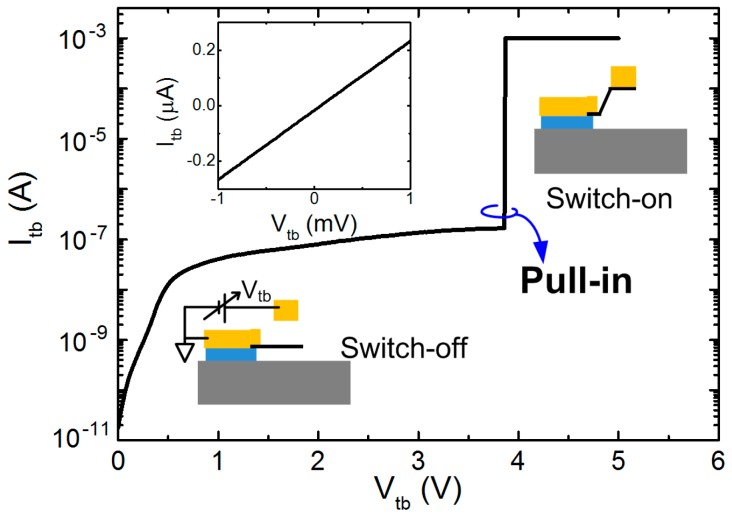
Electrical characterization of the switching performance of a cantilever-type switch. Inset: (**Lower**) two-terminal configuration and switch-off status, (**Right upper**) switch-on status, and (**Left upper**) I-V response after device failure.

A sharp “pull-in” behavior was observed at the *V*_pi_ of 3.8 V (Figure 6). However, this switch was not reversible after the first operation. The linear I-V response was measured between top and bottom electrode indicating that the graphene contacts to the top electrode (left upper inset of Figure 6). Later, the SEM image confirmed that graphene is stuck on the top electrode without any recognized air gap (Figure 5b). 

As is known, the spring constant *k* of a cantilever is:

(2) $$k=\frac{2Ew}{3}{\left(\frac{t}{l}\right)}^{3}$$

which is 48 times lower than the double-clamped beam (Equation (1)). Considering the same deflection, the mechanical restoring force is also 48 times weaker than that in a double-clamped beam of the same dimension. However, interfacial interactions between graphene and the actuation electrode, e.g., van der Waals force, chemical bonds, etc., depend only on the material properties. Hence, in the case of a graphene cantilever, the interfacial force is stronger than its mechanical restoring force, and holds the cantilever on the top electrode even after the retraction of actuation voltage. In a future study, in order to have the reversible switching, surface modification is necessary to reduce these interfacial interactions [6]. 

## 4. Conclusions

We demonstrated the graphene NEM switch with local actuation electrode fabricated via simple fabrication methods. Sharp switching is realized at a low voltage of less than 5 V. These outstanding features benefit future applications of graphene NEM devices for high-performance switching components. In the future, the further lowered pull-in voltage can be achieved through the geometric design. More effort will also be devoted to the improvement of device reliability. For instance, contact interface modification will be utilized to prevent graphene from sticking on contact and increase the number of operation cycles.
